# Combined evaluation of grazing incidence X-ray fluorescence and X-ray reflectivity data for improved profiling of ultra-shallow depth distributions^☆^

**DOI:** 10.1016/j.sab.2014.06.019

**Published:** 2014-09-01

**Authors:** D. Ingerle, F. Meirer, G. Pepponi, E. Demenev, D. Giubertoni, P. Wobrauschek, C. Streli

**Affiliations:** a Atominstitut, Vienna University of Technology, Stadionallee 2, A-1020 Vienna, Austria; b Inorganic Chemistry and Catalysis, Debye Institute for Nanomaterials Science, Utrecht University, Universiteitsweg 99, 3584 CG Utrecht, Netherlands; c MiNALab, CMM-irst, Fondazione Bruno Kessler, Via Sommarive 18, I-38050 Povo, Italy

**Keywords:** GIXRF, XRR, Ultra-shallow junctions, Ultra-shallow implants

## Abstract

The continuous downscaling of the process size for semiconductor devices pushes the junction depths and consequentially the implantation depths to the top few nanometers of the Si substrate. This motivates the need for sensitive methods capable of analyzing dopant distribution, total dose and possible impurities. X-ray techniques utilizing the external reflection of X-rays are very surface sensitive, hence providing a non-destructive tool for process analysis and control.

X-ray reflectometry (XRR) is an established technique for the characterization of single- and multi-layered thin film structures with layer thicknesses in the nanometer range. XRR spectra are acquired by varying the incident angle in the grazing incidence regime while measuring the specular reflected X-ray beam. The shape of the resulting angle-dependent curve is correlated to changes of the electron density in the sample, but does not provide direct information on the presence or distribution of chemical elements in the sample.

Grazing Incidence XRF (GIXRF) measures the X-ray fluorescence induced by an X-ray beam incident under grazing angles. The resulting angle dependent intensity curves are correlated to the depth distribution and mass density of the elements in the sample. GIXRF provides information on contaminations, total implanted dose and to some extent on the depth of the dopant distribution, but is ambiguous with regard to the exact distribution function.

Both techniques use similar measurement procedures and data evaluation strategies, i.e. optimization of a sample model by fitting measured and calculated angle curves. Moreover, the applied sample models can be derived from the same physical properties, like atomic scattering/form factors and elemental concentrations; a simultaneous analysis is therefore a straightforward approach. This combined analysis in turn reduces the uncertainties of the individual techniques, allowing a determination of dose and depth profile of the implanted elements with drastically increased confidence level.

Silicon wafers implanted with Arsenic at different implantation energies were measured by XRR and GIXRF using a combined, simultaneous measurement and data evaluation procedure. The data were processed using a self-developed software package (JGIXA), designed for simultaneous fitting of GIXRF and XRR data. The results were compared with depth profiles obtained by Secondary Ion Mass Spectrometry (SIMS).

## Highlights

• The parameter optimization by curve fitting uses differential evolution (an evolutionary algorithm).
• Implantation profiles are modeled by using the Pearson distribution system.
• The implant distribution profile is discretized to allow calculation similar to a layered sample.
• Total implanted dose, implantation depth and profile shape can be determined nondestructively.

## 1 Introduction

### 1.1 Historical

Grazing incidence x-ray fluorescence (GIXRF) is a surface sensitive technique for the characterization of dopant profiles and thin layers in the nanometer regime on flat and smooth surfaces. The grazing incidence angular dependence of the X-ray fluorescence signal provides information on the depth distribution and total concentration per unit area of the elements in the near surface region. At glancing incidence the predominant part of the incident radiation is reflected and forms – within the limits of coherence – standing waves above the surface while the other part of the field intensity penetrates into the refracting medium as an evanescent wave. In 1954 Parratt [1] first showed in his seminal paper how the modulation of the electromagnetic field can be calculated as a function of the angle of incidence based on reflection, refraction and interference in the vicinity of a flat, sufficiently smooth surface. He also proposed a recursive method for the calculation in the presence of stratified media. Parratt implemented the complete field calculation for the prediction of the reflected part of the beam not only paving the way for X-ray reflectivity (XRR) analysis but also providing the basis for GIXRF analysis. In fact, once the field is known, the number of photoelectric absorption events and hence fluorescence photons can be predicted. This was first demonstrated in 1983 by Becker et al. for a homogeneous sample [2] where an exponentially decaying field excites the atoms in the material. In 1986 Iida et al. [3] used this approach for the study of an Arsenic implanted layer in silicon at a synchrotron radiation facility. In 1991 de Boer [4] published a thorough derivation of the fluorescence emitted from layered samples based on the calculation of the derivative of the Poynting vector through the calculation of the reflection and transmission coefficients at each layer and making use of Parratt's recursive calculation of the electromagnetic field. He showed for the first time the combined measurement and analysis of GIXRF and XRR signals for layered media. Based on deBoer's work, most developments in the theory of GIXRF were achieved during the 1990s, predicting an analytical potential being ahead of the technological capabilities and requirements at that time to produce thin, near surface layers with overall thicknesses in the nanometer region. In 1993 a combination of X-ray techniques for the analysis of thin layered materials was suggested by van den Hoogenhof and de Boer in [5] as Glancing-incidence X-ray analysis (GIXA), they even presented a spectrometer for combined analysis [6]. However, GIXRF being much less sensitive for sample thicknesses of more then a few tens of nanometers it is today, 20 years later, when technologically highly relevant materials with thicknesses of few nanometers are manufactured, that GIXRF attracts new attention. In 2010 Tiwari et al. [7] published the application of combined XRR and GIXRF measurements for the investigation of thin films and multilayered materials. For the calculation of fluorescence intensities in depth profile analysis the authors suggest a numerical integration using the field intensity of an unaltered substrate or layer. This approach is only valid in the dilute regime, i.e. if the change in refractive index due to the dopant is insignificant.

A more general procedure is presented in detail in this work, respecting the physical relevant parameters and avoiding possibly time-consuming numerical integration.

### 1.2 Technical

Measuring the angle dependent fluorescence signal in grazing incidence on an optical flat results in distinct shapes of the recorded angle curve depending on whether an element is present in the bulk material, a thin layer or implant near the surface or a residue on the surface [8], [9]. For implants the curve shape below and near the critical angle is mainly depending on the average implantation depth, whereas the curve at larger angles corresponds to the implanted dose. The measured fluorescence intensity below the critical angle is given by the integration of the product of the implantation profile and the intensity of the exponentially decaying evanescent wave. Unfortunately, an unambiguous de-convolution of the angle dependent fluorescence signal in order to determine the concentration profile is not possible. To overcome this problem, a simultaneous measurement of the intensity of the specular reflected beam was performed. This XRR measurement is done in a classical θ–2θ geometry with one detector rotating on 2θ collecting the reflected photons while simultaneously recording the emitted X-ray fluorescence with a fluorescence detector. The intensity of the reflected beam depends on the electron density, thus on the atomic scattering factors and concentrations. The implantation of an element in a bulk material results in a gradient of the electron density due to the changes in the concentration of the two involved elements and their atomic scattering factors. This change in electron density induces a deviation of the reflected beam intensity curve compared to the bulk. If the concentration of the implanted element in the substrate becomes large and results in a considerable difference of electron densities, the XRR measurement will show Kiessig fringes similar to a layered structure. Thus one can differentiate among different concentration profiles, which are undistinguishable when using only GIXRF data.

## 2 The ambiguity problem

As mentioned in the introduction GIXRF alone is not able to provide an unambiguous solution for concentration profiles in a substrate material. In fact the determination of the concentration requires the solution of an ill-posed inverse problem.

**Fig. 1:**
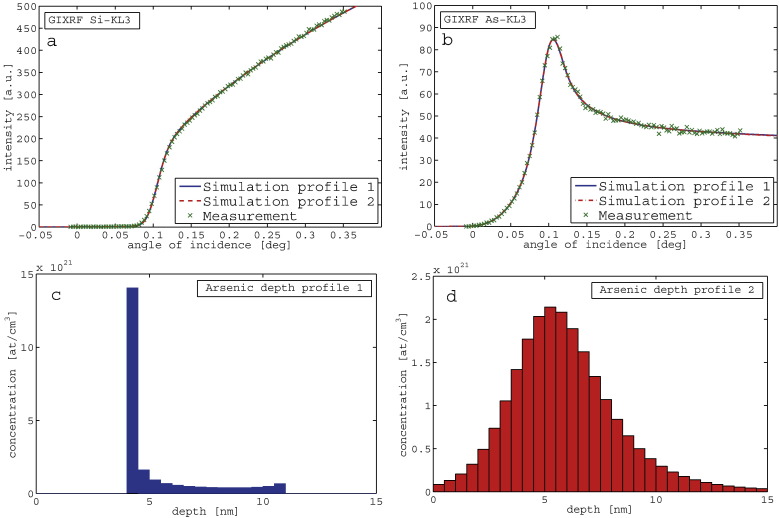
(a) GIXRF Silicon bulk signal (fitted and experimental), (b) GIXRF Arsenic implant signal (fitted and experimental), and (c and d) assumed Arsenic depth profiles for the GIXRF simulation in (a) and (b).

To demonstrate this ambiguity, Fig. 1a and b shows the results of a fitting to GIXRF data using two very different arsenic depth profiles (Fig. 1c and d). The simulation and optimization of the sample parameters was performed following the procedure described in detail in chapter 3. Although the agreement between simulated and measured As and Si GIXRF signals is equally good for both depth profiles, the distribution shown in Fig. 1c is physically unrealistic.

**Fig. 2:**
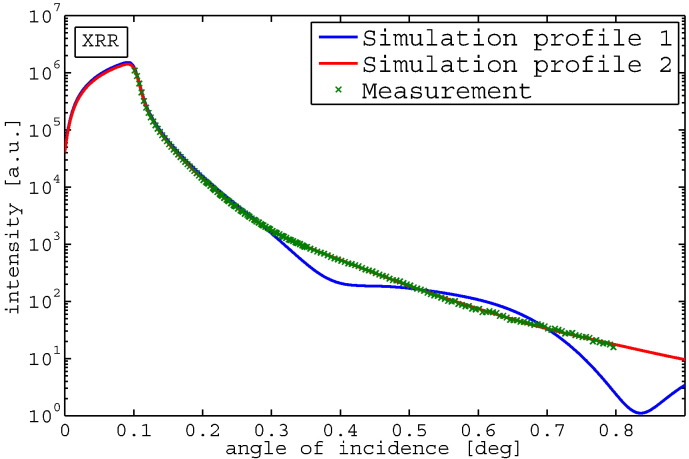
Measured and simulated XRR signal for the profiles shown in Fig. 1c and d.

However, when introducing the XRR measurement and simulations (Fig. 2) based on the profiles in Fig. 1c and d, only the profile shown in 1d leads to an excellent agreement between GIXRF as well as XRR data.

Here it has to be noted that using only the XRR data is also not sufficient to determine the depth profile because also the fit to the collected XRR data has no unique solution [10], [11], [12].

## 3 Methodology

The simulation and fitting of depth profiles is a rather complex task, due to the large number of parameters involved. We therefore provide in the following only a brief, general overview over the approach as implemented in the JGIXA software, further details will be published elsewhere.

### 3.1 Calculation

To allow a successful calculation of XRF and XRR following the approach of de Boer [4] for layered materials, our approach for the determination of the implant distribution profile is based on its discretization in a distinct number of layers. We used a model of 30 layers with a thickness of 0.5 nm each, assuming a maximum profile depth of 15 nm. Thinner layers did not affect the results of the calculation, while thicker layers resulted in changes to the angle dependent GIXRF curve shape.

When the peak concentrations of the depth profiles approach elemental concentrations beyond the dilute regime, that is when the index of refraction starts differing significantly from the value of the substrate, the profile itself starts to severely alter the field intensities in the material. Any data evaluation therefore has to include these modifications of the electrical field using a layer model of the depth profile fitting the key parameters defining each layer.

The values to be evaluated for each layer are the complex refractive index derived from the atomic scattering factors (Henke [13]), the reflected and transmitted amplitudes (derived from the Fresnel equations), the reflected and transmitted fields, the energy flux density calculated via the Poynting vector, the fluorescence emitted by each element in the layer, and the absorption of the emitted radiation on its way out of the sample.

The following equation shows the fluorescence intensity of element a in layer j:

(1) $$I_{\mathrm{aj}}=\frac{\lambda }{\mathrm{hc}}C_{\mathrm{aj}}\rho_{j}\frac{\tau_{\mathrm{a\lambda}}}{\mu_{\mathrm{j\lambda}}}J_{a}w_{a}g_{a}\exp\left(-\sum_{n=1}^{j-1}\frac{\mu_{\mathrm{na}}d_{n}}{\sin\psi_{d}}\right)S\int_{0}^{d_{j}}\mathrm{dz}\left(-\frac{\partial P_{\mathrm{jz}}}{\partial z}\right)\exp\left(-\frac{\mu_{\mathrm{ja}}z}{\sin\psi_{d}}\right)$$

*λ* wavelength of the incident radiation
*h* Planck' s constant
*c* velocity of light in vacuum
*C*_*aj*_ mass fraction of element a in layer j
*ρ*_*j*_ density of layer j
*τ*_*aλ*_ photoelectric absorption coefficient for element a at wavelength *λ*
*μ*_*jλ*_ linear absorption coefficient of the incident radiation in layer j
*J*_*a*_ absorption jump factor for the considered shell
w_*a*_ fluorescence yield
g_*a*_ relative emission rate
*μ*_*na*_ linear absorption coefficient of the considered fluorescence of element a in layer n
d_n_ thickness of layer n
*S* irradiated detected sample area
*ψ*_d_ detector angle
*P*_*jz*_ z component of the Poynting vector
*μ*_*ja*_ linear absorption coefficient of the considered fluorescence of element a in layer j

In addition to the parameters depending on the experimental geometry, the effect of the angular beam divergence has to be taken into account. To model the effect of angular beam divergence the calculated curve is convoluted with a Gaussian point spread function.

As commonly used in XRR data fitting, the XRR curves are fitted starting from slightly below the critical angle to reduce the influence of the direct beam. Furthermore, the XRR data are multiplied by the incident angle to the power of 4 [14] to simplify fitting and to increase the influence of small density variations on the fit [15].

Finally, the goodness of the fits is described by a sum of the reduced chi-square of the individual curves (GIXRF for Si, As and XRR) with i datapoints:

(2) $$\chi_{\mathrm{sum}}^{2}=\sum_{n}\frac{1}{\nu_{n}\cdot x_{n,\max}}\sum_{i}\frac{{\left(x_{n,i,\mathrm{meas}}-x_{n,i,\mathrm{calc}}\right)}^{2}}{x_{n,i,\mathrm{calc}}}$$

where ν is the number of degrees of freedom and *x*_max_ is the maximum calculated value of each curve. This normalization of the chi-squares allows for the combined evaluation of the measurement data, which have a varying number of measurement points and different orders of magnitude.

### 3.2 Optimization

**Fig. 3:**
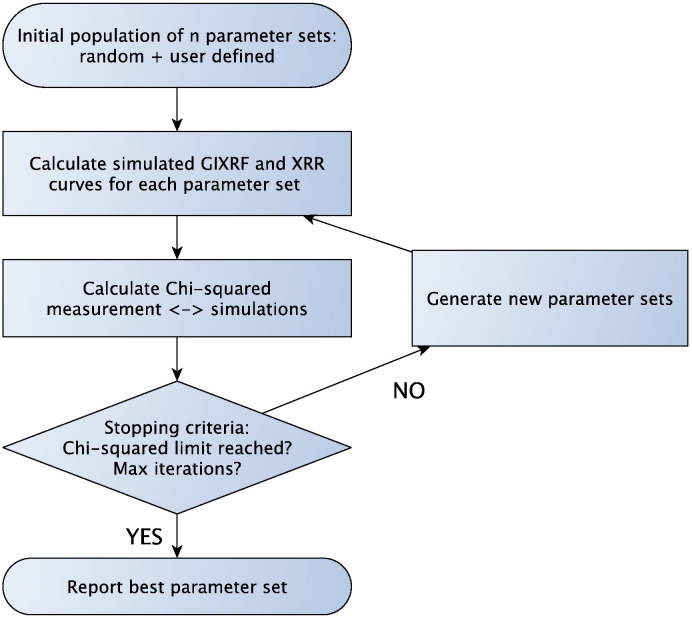
Optimization procedure.

For the optimization procedure two sets of parameters, the experimental setup parameters and the sample parameters have to be defined as initial values. Then the simulated GIXRF and XRR data are calculated for each parameter set and the chi-square between measured and fitted data is determined. If one of the stopping criteria is reached, the best parameter set is reported and no new parameter set is created. A flowchart of the optimization procedure is provided in Fig. 3.

The setup parameters like beam divergence, inspected area, beam size and shape and the relative system sensitivity for fluorescence lines are determined by the measurement and evaluation of reference samples with known composition and are fixed for the evaluation of unknown samples.

The sample parameters consist of shape, depth and total dose of the implantation profile as well as the surface roughness. Because a fit with independent dopant concentrations for each layer would not only be very time consuming but also unrealistic, we limit the number of variable parameters by using the Pearson distribution system [16], [17], which was already used producing good results for the modeling of implantation profiles [18], [19]. This distribution system covers a wide range of shapes. Commonly used distributions like the beta, gamma or normal distribution are subtypes of the Pearson distribution system.

Iterative fitting, using random parameter values as starting points for the fit, performs the optimization. Because the hyper-surface in parameter space for XRR fitting can contain local minima [20], [21], a local optimization algorithm like Levenberg–Marquardt or simplex could produce different results depending on the starting point. Thus global optimization algorithms like direct search, simulated annealing or evolutionary algorithms are required to find the global minimum. Good fitting results have been obtained using genetic algorithms [21], [22], [23] and Wormington et al [24] reported excellent results for XRR and diffraction data fitting utilizing differential evolution [25], which is used in our optimization procedure.

## 4 Experimental

### 4.1 Instrumental setup

The measurements have been performed using the GIXRF vacuum spectrometer at the Atominstitut, as described in [26] retrofitted with a custom/in-house designed XRR module [27]. An Amptek Silicon Drift Detector was used to measure the reflected beam. Though the energy dispersive feature of the SDD was not required, it offers a very low detector noise and a high dynamic range. A Zr filter can be inserted in the beam to reduce the intensity of the primary beam. In this way the dynamic range could be enhanced from 6 orders of magnitude without using the filter, to 8 orders of magnitude when using the filter. More details about the setup can be found in [27].

The primary X-ray source was a Mo 3 kW LFF tube operated at 50 kV/40 mA and a W/C multilayer monochromatized the beam. All spectra were measured for 100 s livetime per angle step, summing to a total measurement time of 3–4 h for each sample.

### 4.2 Samples

For testing the above-described approach, Silicon wafers implanted with Arsenic were used. ^75^As + was implanted in the Czochralski (100) Si wafers (300 mm in diameter) at 0° tilt and twist angles with an Applied Materials Quantum X implanter to produce two sets of samples: one with a constant nominal fluence of 1 × 10^15^ atoms/cm^2^ and implantation energies of 0.5, 2, and 3 keV and one with a constant implantation energy of 2 keV and varying nominal fluence of 1 × 10^14^, 5 × 10^14^ and 1 × 10^15^, (details can be found in Table 1 and [28]). The total dose measurements by INAA, SIMS, and SR-GIXRF are published in [28]. SIMS profiles of the same samples and a model correction procedure involving MEIS data were published in [29]. All SIMS data presented in this work were corrected using this procedure.

**Table 1:** Samples description and dose determined by GIXRF using the JGIXA software in comparison to other techniques.

Sample id	Implant energy [keV]	Dose nominal [at/cm^2^]	Dose JGIXA [at/cm^2^]	Dose NAA [at/cm^2^]	Dose SIMS 500 eV [at/cm^2^]	Dose SIMS 350 eV [at/cm^2^]	Dose SIMS 250 eV [at/cm^2^]
As1	0.5	1.00E + 15	1.11E + 15	1.13E + 15	1.06E + 15	1.01E + 15	9.80E + 14
As3	2.0	1.00E + 15	1.10E + 15	1.13E + 15	1.02E + 15	1.07E + 15	1.11E + 15
As4	3.0	1.00E + 15	1.09E + 15	9.87E + 14	1.06E + 15	1.08E + 15	1.08E + 15
As6	2.0	1.00E + 14	1.17E + 14	1.03E + 14	1.10E + 14	1.00E + 14	1.03E + 14
As7	2.0	5.00E + 14	5.67E + 14	5.59E + 14	5.50E + 14	5.50E + 14	5.46E + 14

As can be seen in Table 1, the JGIXA software is also able to determine the total implanted dose.

## 5 Results and discussion

The following figures report the fit results to the experimental data collected for the samples described above, as obtained by the JGIXA program.

**Fig. 4:**
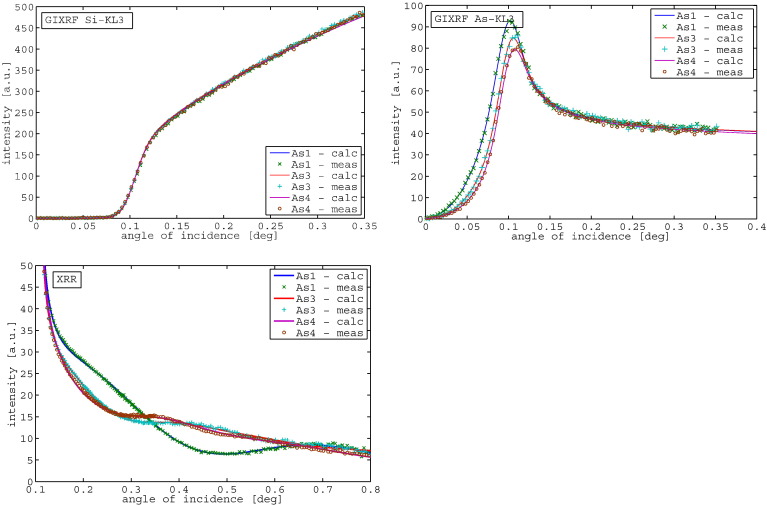
GIXRF of Si, GIXRF of As and XRR for samples with the same dose (1E15), different implantation energy — As1 (0.5 keV), As3 (2 keV), and As4 (3 keV).

**Fig. 5:**
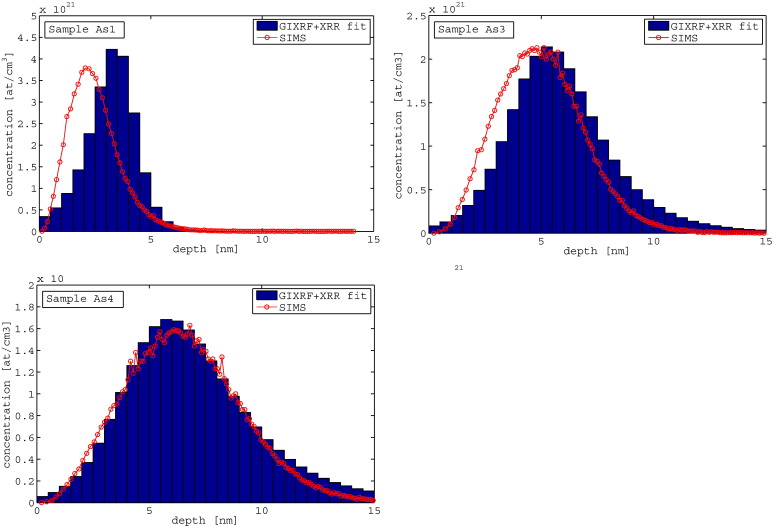
Depth profiles for samples with the same dose (1E15), but different implantation energy — As1 (0.5 keV), As3 (2 keV), As4 (3 keV) — and SIMS data from [29] in red.

Fig. 4 shows the fit results for the Si signal, the As signal and the XRR signal for the samples with the same dose (1E15) but different implantation energy (0.5,2,3 keV), following the sample description in Table. 1. The fits of all curves are in good agreement with the measured data points. In Fig. 5 the implantation profiles obtained from JGIXA in comparison to SIMS data from [29] are shown. The profile of sample As4 (3 keV implantation energy) shows the best correlation with the SIMS profile, while the profile of sample As3 (2 keV implantation energy) shows a slight deviation from the SIMS profile in the direction of increasing depth, but reporting the same total dose. The profile of sample As1 (0.5 keV implantation energy) shows a deviation of 1 nm in the mean implantation depth in the direction to increasing depth and also slight differences in the dose distribution.

**Fig. 6:**
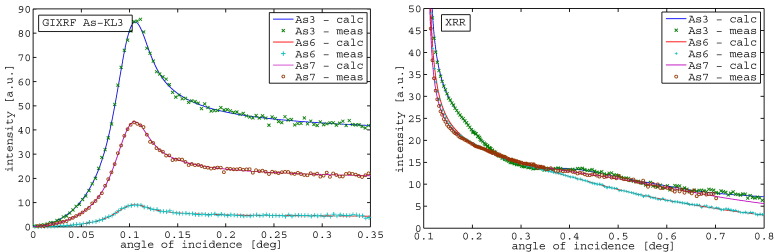
GIXRF-As signal + XRR measurement and fitting for samples with the same implantation energy (2 keV), but different dose — As3 (1E15), As6 (1E14), and As7 (5E14).

The fits for samples with the same implantation energy (2 keV), but different implantation dose (1E15, 1E14, 5E14) are shown in Fig. 6.

**Fig. 7:**
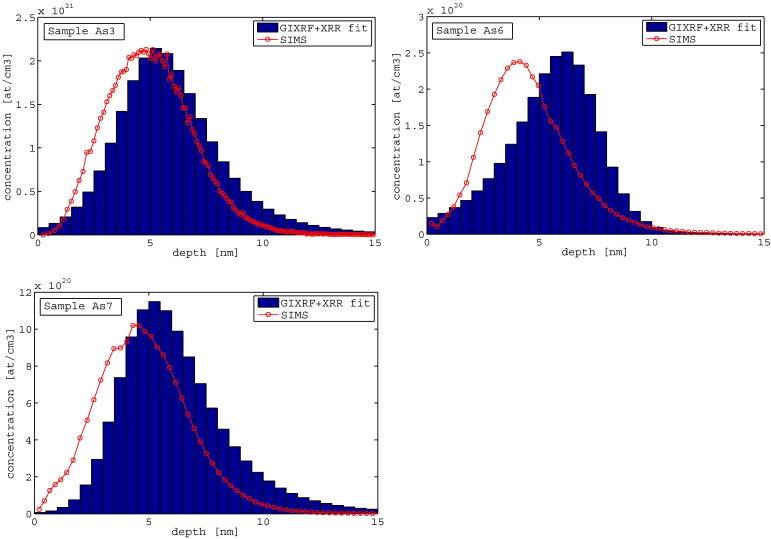
Depth profiles for samples with the same implantation energy (2 keV), but different dose—As3 (1E15), As6 (1E14), As7 (5E14) — and SIMS data from [29] in red.

In Fig. 7 the implantation profiles obtained from JGIXA in comparison with SIMS data from [29] are reported. The profile of sample As3 (1E15 implantation dose) shows the best agreement with the SIMS profile. The profile of sample As6 (1E14 implantation dose) shows a deviation of 1 nm of the mean implantation depth from the SIMS profile in the direction of increasing depth, but the same total dose. The profile of sample As7 (5E14 implantation dose) shows a slight deviation in the mean implantation depth in the direction to increasing depth and also slight differences in the dose distribution.

**Fig. 8:**
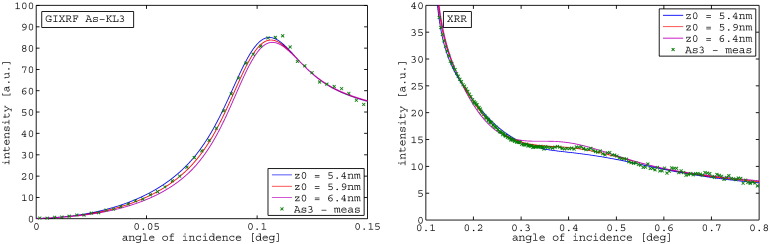
Simulations of depth variations of 0.5 nm around the mean implantation depth in both directions, both the GIXRF As curve as well as the XRR curve show significant discrepancies between simulation and experimental data which are also manifested in an increased chi squared value.

This discrepancy between the results and the SIMS profiles motivated an investigation of the sensitivity of the fits to changes in implantation depth. Fig. 8 shows the results for a change of the mean implantation depth (z0) of 0.5 nm in both directions towards increasing depth or more shallow depth. The GIXRF Arsenic angle curve as well as the XRR data both shows a significant discrepancy between simulation and experimental data for these just 0.5 nm shifts, highlighting the high sensitivity of the presented method.

As a final remark it can be concluded that by using the same layer model to simultaneously calculate the theoretical GIXRF and XRR signals and performing a combined fitting of simulated signals to measured GIXRF and XRR data it becomes possible to find a global minimum in parameter space.

## 6 Conclusions

The combined evaluation of GIXRF and XRR measurements resulted in an improved agreement on profile shape between SIMS and X-ray techniques as well as resolving the ambiguity of fitting only GIXRF data. For deeper implanted samples agreement on depth profile to SIMS data is very good (As4), for very shallow implantation (As1) in a depth of 3–4 nm slight deviations of 0.5–1 nm were observed and have to be investigated further, given that results from SIMS and MEIS also showed minor discrepancies [29]. A sensitivity for changes in the depth of less than 0.5 nm was achieved. The determined values for total implanted dose are in good agreement with other techniques.
